# Occult Primary Pulmonary Adenocarcinoma Presenting With Multistation Nodal Disease: A Case Report

**DOI:** 10.7759/cureus.117246

**Published:** 2026-09-30

**Authors:** Oussama Slimani, Soumiya Samba, Mohammed Bentourqi, Ahmed BenSghier, Soufiane Berhili, Mohamed Moukhlissi, Loubna Mezouar

**Affiliations:** 1 Radiation Oncology, Faculty of Medicine and Pharmacy, Mohammed First University, Oujda, MAR

**Keywords:** mediastinal lymphadenopathy, occult primary lung cancer, pulmonary adenocarcinoma, supraclavicular lymphadenopathy, thyroid transcription factor 1

## Abstract

Occult primary lung cancer is an uncommon presentation in which metastatic disease is histologically and immunophenotypically compatible with a pulmonary origin despite the absence of a detectable lung lesion on CT or fluorodeoxyglucose PET/CT. We report a 55-year-old man who presented with a clinically suspicious left supraclavicular lymph node and an Eastern Cooperative Oncology Group (ECOG) performance status of 1. Excisional lymph node biopsy demonstrated metastatic moderately differentiated adenocarcinoma with glandular, trabecular, and cribriform architecture and mucin production. The tumor showed diffuse strong expression of cytokeratin 7 and thyroid transcription factor 1, whereas cytokeratin 20, CDX2, GATA3, P504S, and cytokeratin 19 were not expressed, supporting a pulmonary origin. Baseline imaging demonstrated hypermetabolic cervical, bilateral supraclavicular, left axillary, and diffuse mediastinal lymphadenopathy without a detectable pulmonary primary or definite visceral or osseous metastases. Epidermal growth factor receptor testing was negative, anaplastic lymphoma kinase expression was absent, and the programmed death-ligand 1 tumor proportion score was below 1%. The patient received six cycles of cisplatin 80 mg/m² and paclitaxel 200 mg/m² intravenously on day 1 of a 21-day cycle. Marked nodal regression after three cycles prompted three additional cycles. At the end of six cycles, imaging showed only small residual nodes, no emerging lung primary, and no new metastatic sites. The patient remained in good general condition with a performance status of 1; documented toxicities were cisplatin-related hypoacusis and grade 1 lower-limb sensory neuropathy attributed to paclitaxel. Maintenance therapy was discussed, but the patient opted for surveillance; subsequent follow-up imaging demonstrated a sustained response without disease progression. This case illustrates the value of integrated histopathology, immunohistochemistry, molecular testing, and serial imaging in diagnosing and managing pulmonary adenocarcinoma with an occult primary site.

## Introduction

Occult primary non-small cell lung cancer (OP-NSCLC) is diagnosed when malignant tissue obtained from a metastatic site is compatible with a pulmonary primary while no primary lung lesion is identified on high-quality thoracic imaging. This presentation is rare and overlaps diagnostically with cancer of unknown primary (CUP), particularly when the disease is first recognized in cervical or supraclavicular lymph nodes [1,2].

When adenocarcinoma is identified in a cervical lymph node, the differential diagnosis includes pulmonary, thyroid, gastrointestinal, pancreaticobiliary, breast, salivary, renal, and genitourinary primaries. Histomorphology must therefore be interpreted together with a focused immunohistochemical panel, molecular testing, and comprehensive imaging [3-7]. We describe a patient with extensive cervical and mediastinal nodal adenocarcinoma, a strongly supportive pulmonary immunophenotype, and no detectable lung primary who achieved a marked radiologic response to cisplatin plus paclitaxel. The extensive multistation nodal distribution, including extrathoracic nodal involvement, also illustrates the therapeutic dilemma between locoregional management and a systemic treatment approach.

## Case presentation

A 55-year-old man presented in July 2025 with progressive left cervical and supraclavicular swelling. He was a chronic smoker who had subsequently stopped smoking. His general condition was preserved, with an Eastern Cooperative Oncology Group (ECOG) performance status of 1 [8]. He reported no associated respiratory, upper aerodigestive, or constitutional symptoms, including cough, dyspnea, hemoptysis, dysphagia, hoarseness, weight loss, or night sweats. A complete otorhinolaryngologic examination revealed no suspicious primary lesion. Physical examination identified a palpable left supraclavicular lymph node with clinically malignant features: it was firm to hard, painless, poorly mobile in relation to the deep planes, and non-inflammatory, without overlying erythema, warmth, or skin ulceration.

Cervical lymph node excision showed lymph node parenchyma infiltrated by a malignant epithelial proliferation arranged in glands, cords, thick trabeculae, and cribriform nests within a fibrous inflammatory stroma. The tumor was moderately differentiated and displayed vesicular nuclei, visible nucleoli, abundant eosinophilic cytoplasm, intraluminal and intracytoplasmic mucin, and vascular tumor emboli. These findings established lymph node metastasis from a moderately differentiated adenocarcinoma (Figure 1).

**Figure 1:**
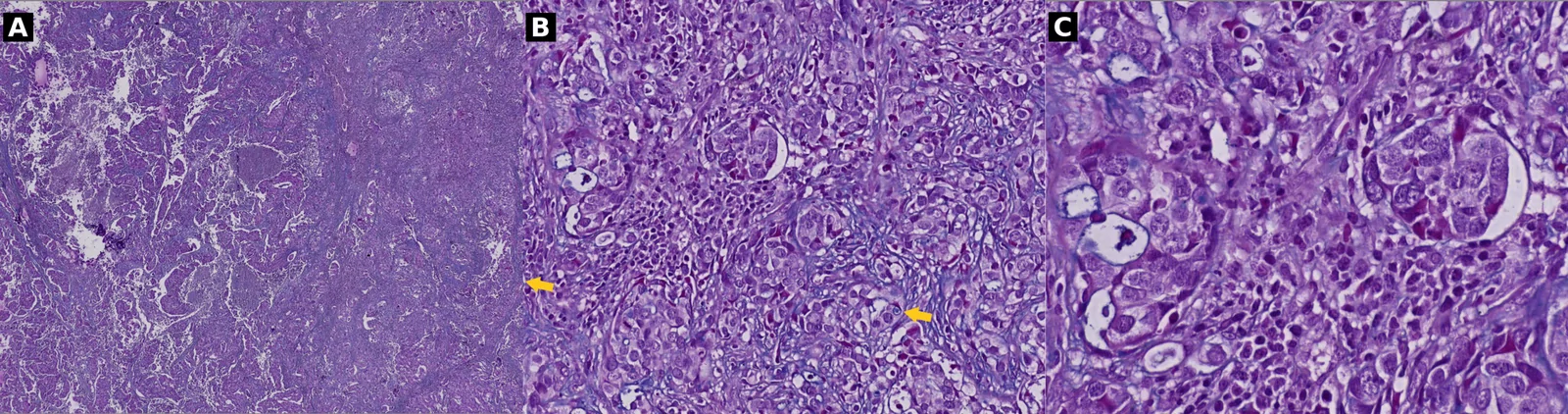
Histopathologic findings in the cervical lymph node. (A) Low-power view showing extensive replacement of the lymph node by moderately differentiated adenocarcinoma arranged in thick trabeculae and cribriform nests within a fibrous inflammatory stroma (H&E-saffron, ×4). (B) Tumor cells arranged in cords, nests, and glands, with intraluminal mucin and intracytoplasmic mucinous vacuoles (yellow arrows; H&E-Alcian blue, ×20). (C) High-power view demonstrating marked nuclear atypia, vesicular chromatin, visible nucleoli, and abundant eosinophilic cytoplasm (H&E-Alcian blue, ×40).

Immunohistochemistry demonstrated diffuse and intense tumor cell staining for cytokeratin 7 and thyroid transcription factor 1. Cytokeratin 20, CDX2, GATA3, P504S, and cytokeratin 19 were not expressed (Table 1).

**Table 1:** Immunohistochemical profile of the cervical lymph node metastasis.

Immunohistochemical marker	Result
Cytokeratin 7 (CK7)	Diffuse and intense positive staining
Thyroid transcription factor 1 (TTF-1)	Diffuse and intense positive staining
Cytokeratin 20 (CK20)	Negative
CDX2	Negative
GATA3	Negative
P504S	Negative
Cytokeratin 19 (CK19)	Negative

In the clinical and morphologic context, this profile strongly favored a pulmonary origin (Figure 2). Predictive testing showed absent anaplastic lymphoma kinase tumor cell staining, programmed death-ligand 1 expression with a tumor proportion score below 1%, and no detectable epidermal growth factor receptor mutation in the examined exons.

**Figure 2:**
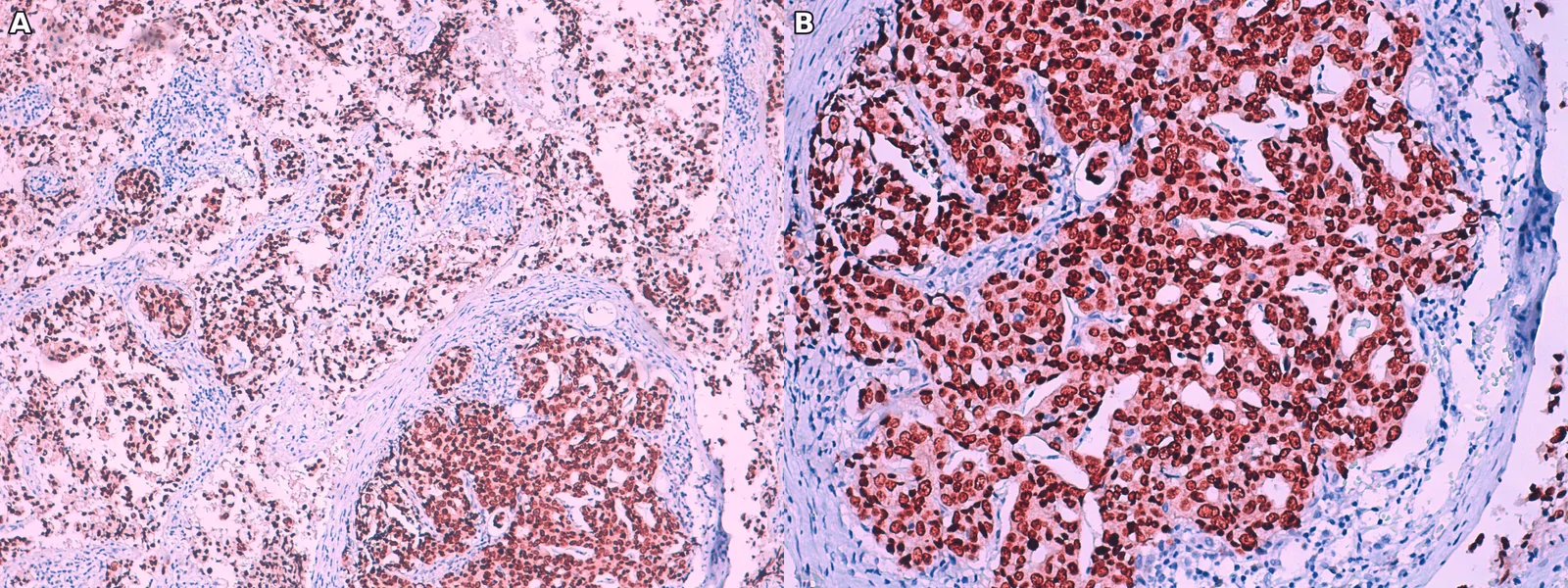
Immunohistochemical staining for thyroid transcription factor 1 (TTF-1). (A) Representative lower-power field and (B) representative higher-power field showing diffuse nuclear staining for TTF-1 in tumor cells.

Baseline contrast-enhanced cranial and thoraco-abdominopelvic CT demonstrated cervical and supraclavicular lymphadenopathy, including a left supraclavicular node measuring up to 18 × 15 mm, together with mediastinal adenopathy. Diffuse bronchial dilatation and bilateral apical emphysematous bullae were present; however, no suspicious pulmonary mass or nodule was identified. No definite visceral or osseous metastatic lesion was demonstrated.

Fluorodeoxyglucose PET/CT showed hypermetabolic left jugular and bilateral supraclavicular lymph nodes, a hypermetabolic left axillary node, and diffuse hypermetabolic mediastinal adenopathy, including a lateroaortic node measuring approximately 30 × 18 mm with a maximum standardized uptake value of 11.47. No hypermetabolic pulmonary primary was detected (Figure 3). There was no evident osseous or visceral secondary localization. Taken together, the morphology, immunophenotype, molecular findings, and imaging supported occult primary pulmonary adenocarcinoma presenting as multistation nodal disease.

**Figure 3:**
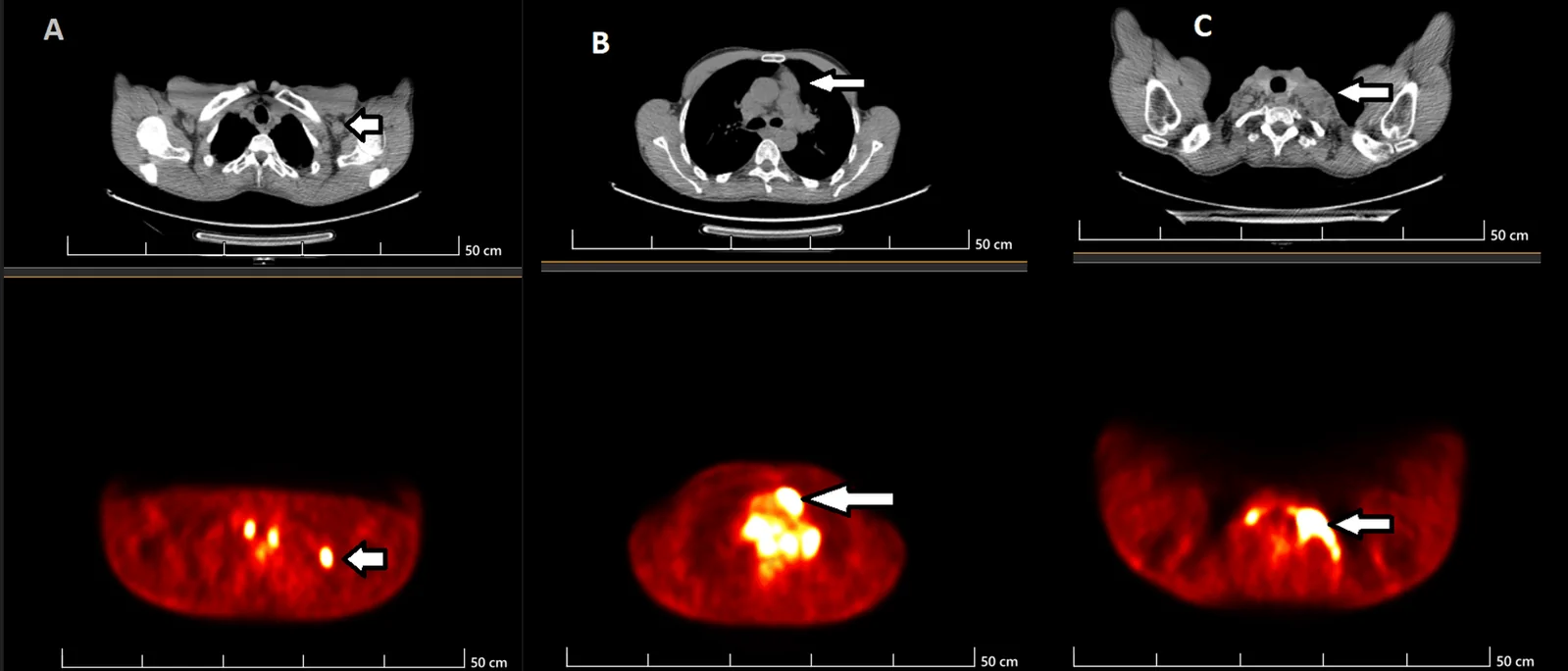
Baseline fluorodeoxyglucose (FDG) PET/CT. Representative axial CT images (upper row) and corresponding PET images (lower row) demonstrate hypermetabolic nodal disease at the axillary (A), mediastinal (B), and cervical (C) levels. White arrows indicate the corresponding involved lymph nodes. No hypermetabolic pulmonary primary was identified on the complete examination.

Given the limited local availability of alternative systemic treatment regimens at the treating institution, cisplatin plus paclitaxel was selected as an available platinum-based doublet. The patient received cisplatin 80 mg/m² intravenously on day 1 plus paclitaxel 200 mg/m² intravenously on day 1, repeated every 21 days. After three cycles, CT demonstrated a marked reduction in the size and number of cervical, supraclavicular, mediastinal, and hilar lymph nodes without the appearance of a pulmonary primary or a new metastatic site. Because of the objective response and preserved clinical condition, three additional cycles of the same regimen were administered, for a total of six cycles.

Final CT in January 2026 confirmed persistent nodal regression, with only small residual mediastinal nodes, including an aortopulmonary-window node measuring approximately 9 × 8 mm. There was no suspicious pleuropulmonary mass, no new visceral or osseous lesion, and no radiologic progression. Representative pretreatment and post-treatment images are shown in Figure 4.

**Figure 4:**
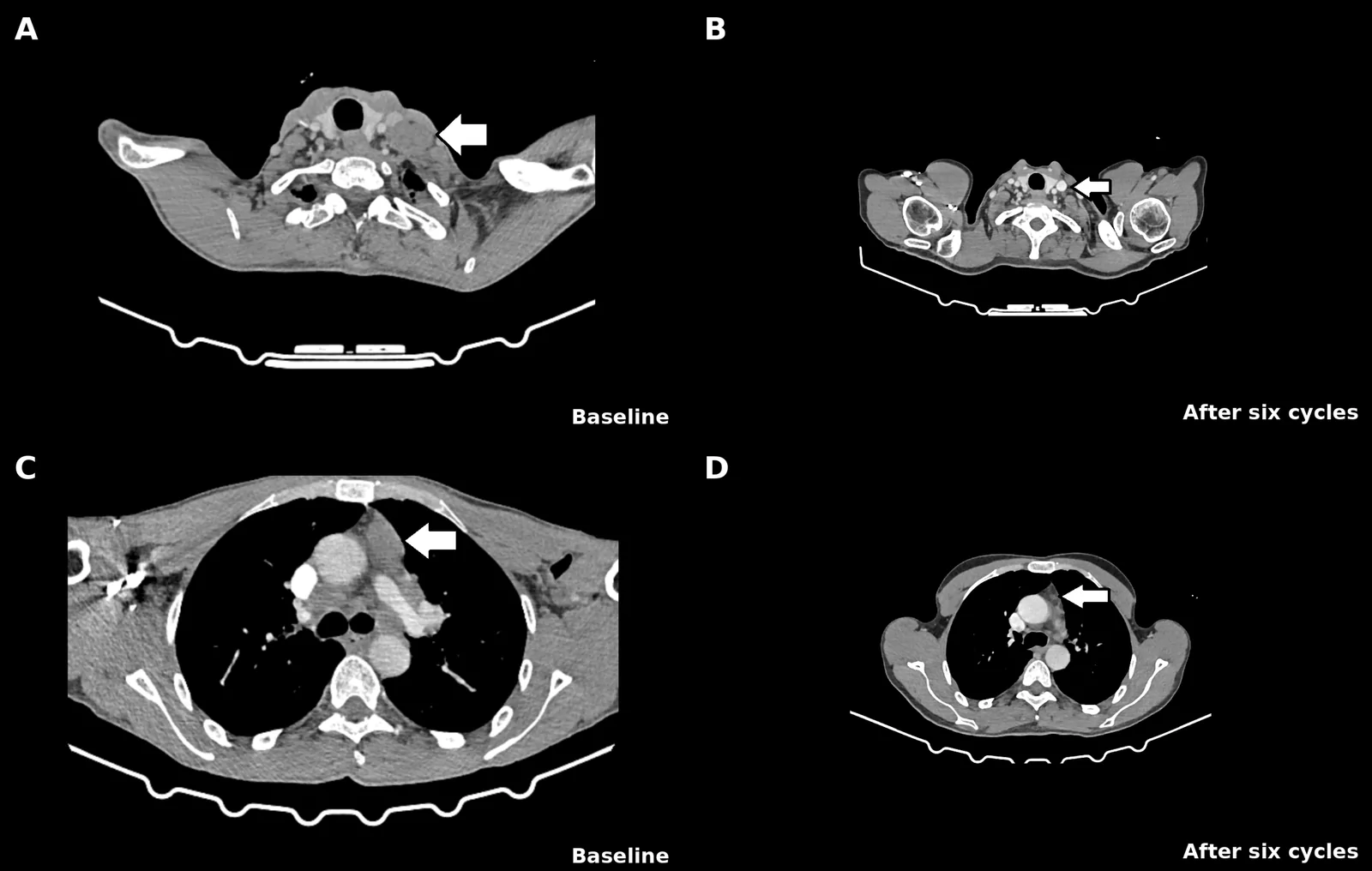
CT comparison before and after chemotherapy. (A) Baseline axial image at the lower cervical/supraclavicular level showing left-sided nodal enlargement. (B) Post-treatment image at a comparable level after six cycles of chemotherapy, demonstrating marked nodal regression. (C) Baseline axial thoracic image showing mediastinal adenopathy at the aortopulmonary-window level. (D) Post-treatment thoracic image showing substantial regression with small residual lymph nodes.

At the end of treatment, the patient remained in good general condition, with an ECOG performance status of 1 [8]. Treatment-related adverse effects consisted of hypoacusis considered compatible with cisplatin-induced ototoxicity and grade 1 sensory peripheral neuropathy of the lower limbs attributed to paclitaxel. Maintenance systemic therapy was discussed and proposed; however, after discussion of the available options, the patient elected to undergo active surveillance. At six months after completion of chemotherapy, he remained clinically well, and surveillance imaging demonstrated a sustained radiologic response, with persistent stability of the residual nodal disease, no evidence of progression, and no emergence of a detectable pulmonary primary.

## Discussion

OP-NSCLC is an uncommon clinicopathologic entity in which metastatic carcinoma has convincing pulmonary differentiation but no primary parenchymal lesion is visible. The largest comparative series identified only 21 occult primary cases among 605 patients treated for stage III NSCLC, illustrating the rarity of the condition [1]. Cervical or supraclavicular presentation further broadens the differential diagnosis because lung cancer can metastasize to head and neck nodal sites but does so far less commonly than to intrathoracic nodes [2]. In the present patient, the combination of gland-forming morphology, mucin production, diffuse TTF-1 and CK7 expression, and absence of markers supporting colorectal, prostatic, urothelial, breast, or other common primaries made pulmonary adenocarcinoma the most coherent diagnosis.

The diagnostic approach should prioritize biopsy of the safest accessible lesion, preservation of tissue, and stepwise pathology rather than indiscriminate use of large immunohistochemical panels. TTF-1 is a sensitive and relatively specific marker for pulmonary adenocarcinoma when interpreted with morphology, while CK7 is sensitive but less specific; neither marker should be used in isolation [3,4]. Once a pulmonary phenotype is established, contemporary recommendations support broad molecular profiling in advanced nonsquamous NSCLC because actionable alterations extend beyond EGFR and ALK to ROS1, BRAF, MET exon 14, RET, NTRK, KRAS G12C, ERBB2, and other targets [5,6]. The limited panel in this case is therefore an important limitation, and comprehensive next-generation sequencing would be appropriate if tissue remains available or at disease progression.

Management depends on the anatomic extent of disease. When occult primary NSCLC is confined to mediastinal or hilar regional nodes and can be encompassed within a definitive treatment field, treatment analogous to that for unresectable stage III NSCLC with concurrent chemoradiotherapy may be appropriate. In selected thoracic-node-only occult cases, definitive radiotherapy has produced favorable long-term outcomes [1]. For unresectable stage III NSCLC without progression after chemoradiotherapy, consolidation durvalumab is the current standard and yields a durable survival benefit [9]. This strategy cannot be automatically extrapolated to the present case because the patient had cervical, bilateral supraclavicular, and left axillary involvement in addition to mediastinal disease, favoring systemic management rather than a thoracic-only curative radiation approach.

For disseminated nodal disease with a strongly supported pulmonary origin, treatment should generally follow advanced nonsquamous NSCLC principles while also considering CUP guidance [7,10]. In current practice, comprehensive molecular testing should precede systemic therapy whenever feasible. In patients without actionable driver alterations, pembrolizumab plus pemetrexed and platinum is a standard first-line option irrespective of PD-L1 expression, including tumors with PD-L1 expression below 1%; five-year results from KEYNOTE-189 confirm a sustained survival advantage over chemotherapy alone [10,11]. When immunotherapy is unavailable, contraindicated, or not selected, a platinum-based doublet remains an evidence-based cytotoxic option.

The cisplatin-paclitaxel regimen used in this patient has established activity in advanced NSCLC. Randomized trials demonstrated that platinum doublets, including cisplatin plus paclitaxel, produce comparable overall efficacy to other active regimens, and paclitaxel-cisplatin has historically yielded meaningful response rates in fit patients [12,13]. Platinum-taxane regimens have also been widely used as empirical treatment for unfavorable CUP. Prospective CUP studies show that these regimens are active, although outcomes remain heterogeneous, and molecularly assigned site-specific therapy has not consistently improved survival over empirical carboplatin-paclitaxel in randomized data [14,15]. The major nodal response in this case supports treatment sensitivity but does not, by itself, prove the pulmonary origin.

Toxicity monitoring is essential when cisplatin and paclitaxel are used. The patient developed hypoacusis consistent with cumulative cisplatin ototoxicity and grade 1 sensory neuropathy consistent with taxane toxicity. Continued audiologic assessment, neurologic examination, renal monitoring, and reassessment of cumulative treatment burden are appropriate before any future platinum- or taxane-containing therapy. At relapse, treatment selection should be driven by repeat staging, comprehensive molecular profiling, prior exposure, residual toxicity, and access to immunotherapy or targeted therapy.

Prognosis in occult-primary pulmonary adenocarcinoma is heterogeneous and depends more on disease distribution, performance status, tumor biology, and treatment responsiveness than on the absence of a visible lung mass alone. Thoracic-node-only occult NSCLC may have a more favorable course than conventional stage III disease: in the Romesser series, five-year overall survival was 61.6% for occult-primary cases versus 15.2% for known-primary stage III NSCLC [1]. These results should not be generalized to patients with extrathoracic nodal spread. In contemporary unfavorable CUP cohorts, median overall survival remains approximately 10 months, and poor performance status is consistently associated with worse outcomes [16]. Favorable features in the present patient include a performance status of 1, absence of visceral and osseous metastases, and a marked radiologic response; adverse features include multistation extrathoracic nodal involvement, absence of an actionable alteration on the limited panel, and PD-L1 expression below 1%. Long-term serial imaging is required because an occult primary may later become visible and because the durability of the response is not yet known.

This report has limitations. The anatomic primary site was not demonstrated, the precise TNM stage is difficult to assign without a visible primary and exact mapping of every cervical nodal level, and the molecular workup was incomplete by current standards. Nevertheless, the concordance of morphology, immunophenotype, imaging, and treatment response supports the working diagnosis of occult-primary pulmonary adenocarcinoma. Multidisciplinary review remains essential, especially when deciding between definitive locoregional therapy and systemic treatment.

## Conclusions

Metastatic adenocarcinoma involving cervical, supraclavicular, axillary, and mediastinal lymph nodes may be of presumed pulmonary origin even when no primary pulmonary lesion is visible on computed tomography or fluorodeoxyglucose positron emission tomography/computed tomography. Establishing the most likely primary site requires integration of lymph-node morphology, a judicious immunohistochemical panel, appropriate molecular testing, and serial imaging.

Management should be individualized according to disease distribution. Thoracic-node-only presentations may be candidates for definitive chemoradiotherapy followed by consolidation immunotherapy, whereas multistation extrathoracic nodal disease may favor a systemic treatment approach according to advanced nonsquamous NSCLC or unfavorable CUP principles. In this patient, six cycles of cisplatin plus paclitaxel produced marked and sustained nodal regression with preserved performance status. Ongoing surveillance and toxicity follow-up remain warranted, and broader genomic profiling should be considered, if feasible, particularly in the event of disease progression.

## References

[1] Romesser PB, Bardash Y, Buonocore D (2019). Outcomes of stage III NSCLC with occult primary vs. known primary lesions. Lung Cancer.

[2] Capodiferro S, d'Amati A, Barile G (2023). Metastatic lung cancer to the head and neck: a clinico-pathological study on 21 cases with narrative review of the literature. J Clin Med.

[3] Yatabe Y, Dacic S, Borczuk AC (2019). Best practices recommendations for diagnostic immunohistochemistry in lung cancer. J Thorac Oncol.

[4] Gurda GT, Zhang L, Wang Y (2015). Utility of five commonly used immunohistochemical markers TTF-1, Napsin A, CK7, CK5/6 and P63 in primary and metastatic adenocarcinoma and squamous cell carcinoma of the lung: a retrospective study of 246 fine needle aspiration cases. Clin Transl Med.

[5] Lindeman NI, Cagle PT, Aisner DL (2018). Updated molecular testing guideline for the selection of lung cancer patients for treatment with targeted tyrosine kinase inhibitors: guideline from the College of American Pathologists, the International Association for the Study of Lung Cancer, and the Association for Molecular Pathology. J Thorac Oncol.

[6] Mosele MF, Westphalen CB, Stenzinger A (2024). Recommendations for the use of next-generation sequencing (NGS) for patients with advanced cancer in 2024: a report from the ESMO Precision Medicine Working Group. Ann Oncol.

[7] Krämer A, Bochtler T, Pauli C (2023). Cancer of unknown primary: ESMO Clinical Practice Guideline for diagnosis, treatment and follow-up. Ann Oncol.

[8] Oken MM, Creech RH, Tormey DC, Horton J, Davis TE, McFadden ET, Carbone PP (1982). Toxicity and response criteria of the Eastern Cooperative Oncology Group. Am J Clin Oncol.

[9] Spigel DR, Faivre-Finn C, Gray JE (2022). Five-year survival outcomes from the PACIFIC trial: durvalumab after chemoradiotherapy in stage III non-small-cell lung cancer. J Clin Oncol.

[10] Hanna NH, Schneider BJ, Temin S (2020). Therapy for stage IV non-small-cell lung cancer without driver alterations: ASCO and Ontario Health joint guideline update. J Clin Oncol.

[11] Garassino MC, Gadgeel S, Speranza G (2023). Pembrolizumab plus pemetrexed and platinum in nonsquamous non-small-cell lung cancer: five-year outcomes from the phase 3 KEYNOTE-189 study. J Clin Oncol.

[12] Schiller JH, Harrington D, Belani CP (2002). Comparison of four chemotherapy regimens for advanced non-small-cell lung cancer. N Engl J Med.

[13] Bonomi P, Kim K, Fairclough D (2000). Comparison of survival and quality of life in advanced non-small-cell lung cancer patients treated with two dose levels of paclitaxel combined with cisplatin versus etoposide with cisplatin: results of an Eastern Cooperative Oncology Group trial. J Clin Oncol.

[14] Huebner G, Link H, Kohne CH (2009). Paclitaxel and carboplatin vs gemcitabine and vinorelbine in patients with adeno- or undifferentiated carcinoma of unknown primary: a randomised prospective phase II trial. Br J Cancer.

[15] Hayashi H, Kurata T, Takiguchi Y (2019). Randomized phase II trial comparing site-specific treatment based on gene expression profiling with carboplatin and paclitaxel for patients with cancer of unknown primary site. J Clin Oncol.

[16] Boys EL, Gao B, Grimison P, Sutherland S, MacKenzie KL, Reddel RR, Liu J (2024). Retrospective analysis of clinical characteristics and outcomes of patients with carcinoma of unknown primary from three tertiary centers in Australia. Cancer Med.

